# Prediction of Thermal Conductivities of Rubbers by MD Simulations—New Insights

**DOI:** 10.3390/polym14102046

**Published:** 2022-05-17

**Authors:** Aleksandr Vasilev, Tommy Lorenz, Cornelia Breitkopf

**Affiliations:** Chair of Technical Thermodynamics, Technische Universität Dresden, 01069 Dresden, Germany; tommy.lorenz@tu-dresden.de (T.L.); cornelia.breitkopf@tu-dresden.de (C.B.)

**Keywords:** molecular dynamics simulations, Green–Kubo method, non-equilibrium molecular dynamics simulations, force fields, rubber, polyisoprene, degree of crosslinking, thermoplastic polyurethane, thermal conductivity

## Abstract

In this article, two main approaches to the prediction of thermal conductivities by molecular dynamics (MD) simulations are discussed, namely non-equilibrium molecular dynamics simulations (NEMD) and the application of the Green–Kubo formula, i.e., EMD. NEMD methods are more affected by size effects than EMD methods. The thermal conductivities of silicone rubbers in special were found as a function of the degree of crosslinking. Moreover, the thermal conductivities of thermoplastic polyurethane as function of the mass fraction of soft segments were obtained by those MD simulations. All results are in good agreement with data from the experimental literature. After the analysis of normalized heat flux autocorrelation functions, it has been revealed that heat in the polymers is mainly transferred by low-frequency phonons. Simulation details as well as advantages and disadvantages of the single methods are discussed in the article.

## 1. Introduction

The development of new types of polymer-based composites requires the knowledge of the properties of their constitutive parts. For instance, for the modeling of heat transfer in a bulk material by finite element methods, the specific heat capacity as well as the thermal conductivity have to be known. These properties can be defined experimentally, as well as, nowadays, by theoretical approaches as molecular dynamics (MD) simulations. There are two main approaches to the prediction of thermal conductivities by MD simulations, namely non-equilibrium molecular dynamics (NEMD) and equilibrium molecular dynamics (EMD) simulation, e.g., application of the Green–Kubo formula. Thermal conductivity calculations by NEMD and EMD methods are equivalent to each other when simulation times and sizes of simulated systems are big enough [1].

EMD or the Green–Kubo method calculates all components of tensors of the thermal conductivity and provides information regarding the contribution of different types of interaction to the thermal conductivity but takes much more time for the equilibration of a simulated system [2]. The method is based on the fluctuation-dissipation theorem [2]. In comparison with non-equilibrium methods, the EMD method depends less on sizes of the simulated systems [3,4].

The EMD method has been widely applied for polymers. For example, the thermal conductivity of crystalline polyethylene has been investigated depending on the uniaxial strain [5]. It has been revealed that the thermal conductivity increases significantly in the axial direction, whereas in the radial direction it decreases with strain. In another example, the influence of the number of cross-links between polymer chains by sulfur bridges to the thermal conductivity of cis-1,3-polyisoprene has been determined [6]. In this study, interactions between the atoms was described by an united atom force field. Moreover, the thermal conductivity as function of uniaxial strain was found. The results are in good agreement with experimental data. Similar results for the thermal conductivity have been found in cross-linked epoxy resin and parallel-linked epoxy resin under uniaxial tensile strain in intra-chain directions [7]. It has been shown by the simulations that the thermal conductivity increases along the direction of the chains, whereas in inter-chain directions it decreases. The thermal conductivity of natural rubber has also been calculated using the Green–Kubo method [8]. Interactions between the atoms have been described by an adaptive inter-molecular reactive empirical bond order (AIREBO) potential. This force field considers each atom explicitly. It has been observed that the thermal conductivity is higher in the direction of the chains in ordered systems. A combination of EMD and finite element methods has been used to predict the thermal conductivity of pitch-based carbon fibers [9]. The results are comparable with experimental data.

NEMD methods are based on obtaining a constant temperature gradient and the heat flux inside a modeled system and the application of the Fourier’s heat transfer equation, when the type of heat transfer is diffusive. In these simulations, the system is divided into slabs, and two of them are defined as hot and cold slabs, which serve as hot and cold plates in a real experiment (for example, the guarded hot plate and heat flow meter methods [10]). There are three algorithms in NEMD simulations that can be used to obtain the steady state regime inside the system.

In the reverse non-equilibrium molecular dynamics (RNEMD) simulation method [11], the heat flux inside a system is imposed by a periodic velocity exchange between the “hottest” atom in the cold slab and the “coldest” atom in the hot slab, which have the same mass. The velocity exchange triggers the heat flux from the hot to the cold slab, which leads to a temperature gradient inside the system.

The period of the velocity exchange influences the temperature gradient. For instance, low values lead to high temperature gradients, and the steady-state regime is reached faster than at high values [12], but, on the other hand, at low values, a modeled substance can be in the periodic box at different phases, for example, in liquid and solid states. In addition, high values of the period of the velocity exchange can lead to big errors [13], whereas low values cause velocity drifting [14], and the thermal conductivity can be affected by the wave motion heat conduction [15]. For the elimination of such cases, one needs to find the optimal period of the velocity exchange.

In the eHEX approach [16], the steady-state regime is achieved by the extraction of energy from the “cold” slab and adding the same amount of energy to the “hot” slab. In the eHEX and RNEMD methods, the heat flux is established before the temperature gradient, whereas in the third method, which is based on application of the Langevin thermostat [17], the temperature gradient is achieved first and after that the heat flux is calculated.

With the NEMD method, the thermal conductivity is calculated only in one direction, and it strongly depends on the sizes of simulated systems [3,4,11]. The method has been widely used for polymers. For instance, the thermal conductivities of polystyrene, stretched polystyrene, mixtures of polystyrene and carbon dioxide have been investigated as functions of temperature and pressure using RNEMD [18]. These results are in good agreement with experimental data.

In comparison to the EMD approach, NEMD methods can be used for the calculation of interfacial thermal resistance between materials [19,20,21,22].

Size effects in amorphous polymers were investigated in Ref. [23]. It has been noticed that thermal conductivites obtained by the EMD method for bulk polymers are in good agreement with experimental data.

Due to their outstanding mechanical properties, silicone rubbers are widely used in soft robotics [24,25,26,27]. Thermal conductivities of amorphous and crystalline silicone rubbers have been investigated by NEMD methods [28]. Deriving the influence of the degree of crosslinking on to the thermal conductivities and mechanical properties of silicone rubbers is important for finding an optimal chemical composition for a concrete application of new rubber, and the knowledge of the thermal conductivity is vital for macro-scale modeling of the rubbers by FEM [29,30,31].

The influence of graphene on the thermo-mechanical properties of TPU-based composites have been investigated [32,33]. It has been revealed that, with an increase in the mass-fraction of graphene, the thermal conductivity and glass transition temperature of TPU-based composites increase.

Due to the composition of TPU, which consists of hard and soft segments (see Simulation Details), different thermo-mechanical properties of TPU can be achieved by adjusting the proportion of the segments. For a first evaluation, those tendencies can be investigated by MD simulations, which was done in this research, based on the optimized force field used [10,34]. The results of thermal conductivities are important for the modeling of fibers based on TPU by FEM [35,36].

Different rubber structures were investigated, and their thermal properties are discussed in detail by MD with respect to the size effects and the appropriate MD method (NEMD, EMD) for simulation.

## 2. Simulation Details

**Figure 1:**
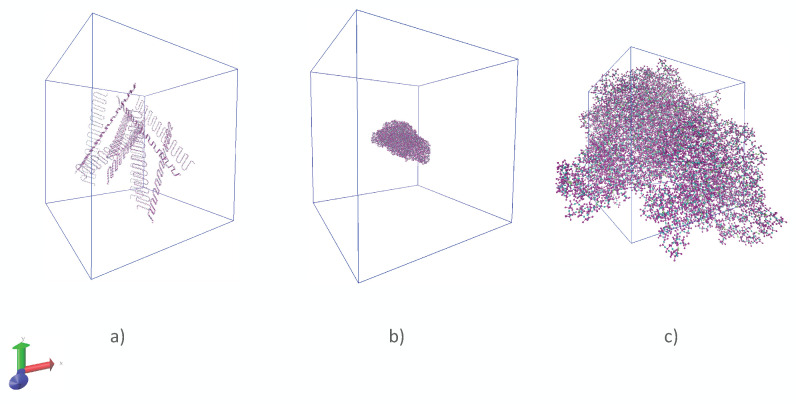
(**a**) Randomly distributed chains of silicone rubber in the periodic supercell; (**b**) chains of silicone rubber after crosslinking; (**c**) structure of silicone rubber before calculation of the thermal conductivity.

For the modeling of silicone rubbers, polymeric chains were created via the Moltemplate [37] software. Every chain contained 418 monomer units. After that, ten polymeric chains were randomly distributed in a periodic supercell (see Figure 1) via the Packmol [38] software. The chains were crosslinked in an NVT ensemble. When such a model for a silicone rubber was obtained, it was simulated in an NPT ensemble until the equilibration of its density. The type of the crosslinking bridge was the same as in Ref. [29]. In general, models of silicone rubbers with a degree of crosslinking of 0%, 5%, 12.9%, and 13% were considered. The force field parameters were taken from OPLS-AA force field.

Before calculating the thermal conductivity of silicone rubbers by the Green–Kubo method, the system was modeled in an NVT ensemble for 100 ps with a time step 1 fs for the equilibration of its temperature. Finally, the heat flux autocorrelation function was calculated in an NVE ensemble for 3 ns with a correlation time of 3 ps. The simulation details are similar to those used in Ref. [29], where they are provided in more detail.

Equation (1) was used as definition for the degree of crosslinking.

(1) $$\mathrm{DC}=\frac{2N_{\mathrm{CL}}}{N_{\mathrm{mono}}}\cdot 100\%,$$

where $N_{\mathrm{CL}}$ and $N_{\mathrm{mono}}$ denote the total numbers of crosslink bridges and monomer units, respectively.

**Figure 2:**
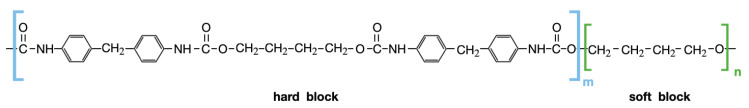
Chemical structure of TPU used in the MD simulations.

Chemical structures of the modeled thermoplastic polyurethane (TPU) chains are shown in Figure 2. A similar chemical structure of TPU has also been considered in Ref. [39].

To investigate the influence of the number of soft segments on the thermal conductivity, six types of TPU systems were considered for the first time. In each system, a polymeric chain was constructed with 40 segments. For every type of a such system, the number of hard and soft segments was varied (see Table 1. The force field parameters were taken from the OPLS-AA force field.

**Figure 3:**
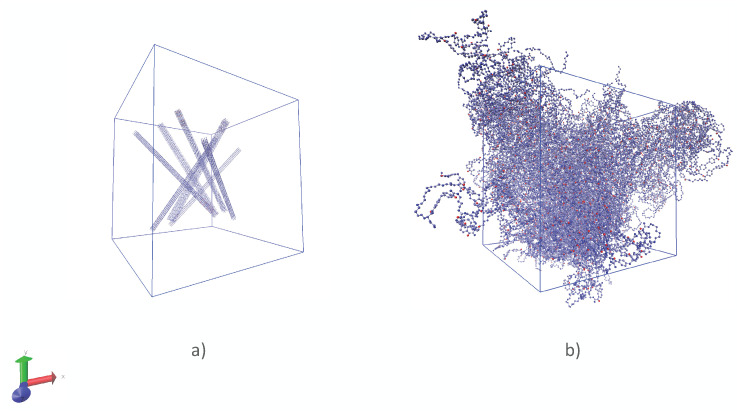
(**a**) Chains of TPU randomly distributed in the periodic supercell; (**b**) structure of TPU before calculation of the thermal conductivity.

Polymeric chains were randomly distributed in the periodic supercell using the Packmol software (see Figure 3). After that, the chains were modeled in an NVT ensemble with a Langevin thermostat. In the next step, the system was heated to high pressures and slowly cooled down until normal conditions in an NPT ensemble, several times, to achieve a density close to the density at normal conditions. Finally, temperature, density and energy of a system were equilibrated at normal conditions.

The models were simulated in an NVE ensemble, where at each time step, a heat flux tensor was recorded, which was used for the calculation of the heat-flux autocorrelation function. The thermal conductivity of a model was determined via the Green–Kubo formula.

In the Green–Kubo formula thermal conductivity of amorphous bulk material is calculated as

(2) $$\lambda =\frac{V}{3k_{B}T^{2}}\int_{0}^{\infty }\;<\vec{J}\left(0\right)\vec{J}\left(t\right)>dt,$$

where $\vec{J}$ is the heat flux, which is defined by the equation from Ref. [40].

For a comparison of EMD and NEMD methods in describing rubbers, the structures of amorphous polyisoprene have been constructed via the Moltemplate and the Packmol software. In the case of EMD simulations, three structures with 12,000, 24,000, and 48,000 atoms were considered. They were modeled for 100 ps in an NPT ensemble to equilibrate the density. After that, the energy and temperature of the structures were equilibrated in an NVT ensemble for 100 ps. Finally, in an NVE ensemble for every time step, a heat flux autocorrelation function was calculated for each correlation time interval, which was equal to 3 ps. The structures were modeled in the micro-canonical ensemble for 3 ns, and thus the thermal conductivity was calculated for 1000 time intervals. For every correlation time interval, it was averaged for the last 0.5 ps.

**Figure 4:**
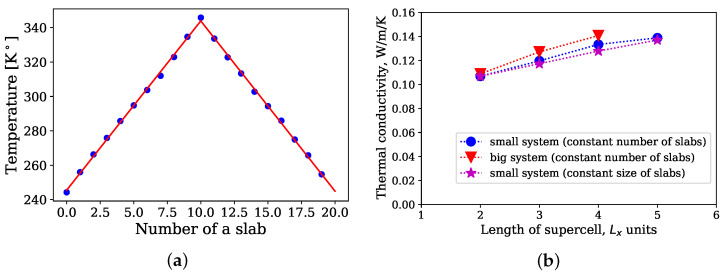
(**a**) Temperature profile inside the periodic supercell; (**b**) dependency of thermal conductivities on the length of the periodic supercell.

The dependency of thermal conductivities on size effects was investigated via the RNEMD method. Three types of systems were considered. In the first type (see magenta stars in Figure 4) all structures consisted of 20 slabs. For every structure the size of the periodic supercell was increased in the direction of the *z* axis for one cubic cell ($L_{x}$ = $L_{y}$ = $L_{z}$) which contained 12,000 atoms. This procedure was done for every type of structure. In the second kind of systems (see red triangles in Figure 4) the total number of atoms in one cubic cell was equal to 24,000 and number of slabs was equal to 20 as structures of the first type of the system. Finally, the third type represents a situation, when the number of slabs changes, whereas sizes of the slabs remain constant. One cubic cell contains 12,000 atoms. The force field parameters were the same as in ref. [10].

All NEMD simulations were performed for 1.8 ns with a time step 1 fs. Velocities between the hottest atom of the cold slab and the coldest atom of the hot slab were exchanged every 450 time steps. For each slab, the average temperature was calculated using data points from the last 1.35 ns of the simulation time. As an example, in Figure 4a the temperature profile for the system of the first type with length of the periodic supercell $L_{z}$ = 5$L_{x}$ is shown. The average temperature from each slab was used to obtain by linear fitting the temperature profile.

## 3. Results and Discussion

**Figure 5:**
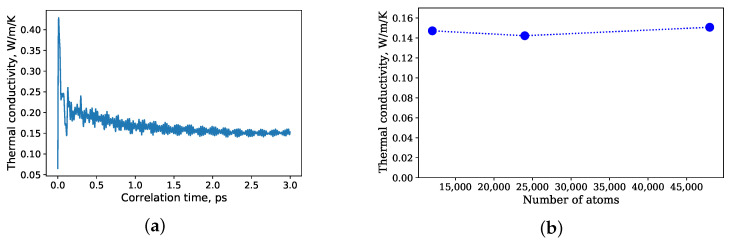
(**a**) Thermal conductivity as a function of correlation time; (**b**) thermal conductivity as a function of the total number of simulated atoms by EMD simulations.

Figure 5a,b illustrate the dependency of the thermal conductivity on the number of atoms. EMD simulations with 12,000, 24,000, and 48,000 atoms resulted in thermal conductivities of 0.147 W/m/K, 0.142 W/m/K and 0.151 W/m/K, respectively. Thus, a system with 12,000 atoms was sufficient to obtain the thermal conductivity for natural rubber at normal conditions.

The dependency of the thermal conductivity on the length of the periodic supercell for the NEMD simulations is shown in Figure 4b. The thermal conductivity for all three cases increases with an increase in the length, which indicates that the sizes of the periodic supercells were not sufficient for a calculation of thermal conductivities of natural rubber. In comparison with the Green–Kubo method, NEMD simulations more affected by size effects. For instance, 12,000 united atoms were sufficient for calculation of the thermal conductivity, whereas for the NEMD simulations, even 48,000 united atoms in the periodic supercell ($L_{z}$ = 4$L_{x}$, $L_{x}$ = $L_{y}$) were not enough.

**Figure 6:**
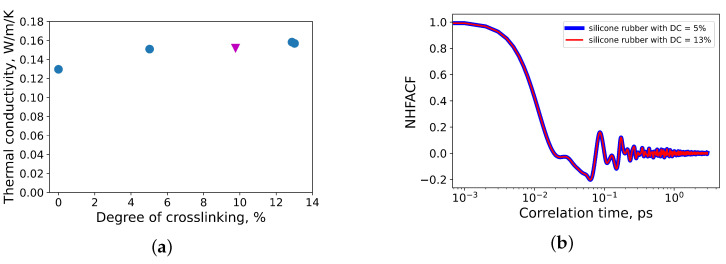
(**a**) Thermal conductivity of silicone rubber as a function of the degree of crosslinking. For comparison, the data point corresponding to the degree of crosslinking 9.8 % (red triangle) from Ref. [29] is shown; (**b**) Normalized heat flux autocorrelation function (NHFACF) as function of correlation time.

The dependency of thermal conductivity of silicone rubber on the degree of crosslinking is presented in Figure 6a. The thermal conductivity linearly increases with an increase in the degree of crosslinking. This tendency has been observed in other polymeric systems by MD simulations [41,42,43,44] and by the network model [45,46].

Quantitative results for thermal conductivities by MD simulations are close to experimental data (≈0.17 W/m/K [47]). The first minimum of the normalized heat flux autocorrelation function (see Figure 6) is located roughly at 63 fs, which corresponds to a wave number $\bar{\lambda }$≈ 84 ${\mathrm{cm}}^{-1}$. Applying the harmonic oscillator model proposed in [6] at normal conditions, heat in silicone rubber is transferred via the deformation of the monomers along the polymer backbone.

**Figure 7:**
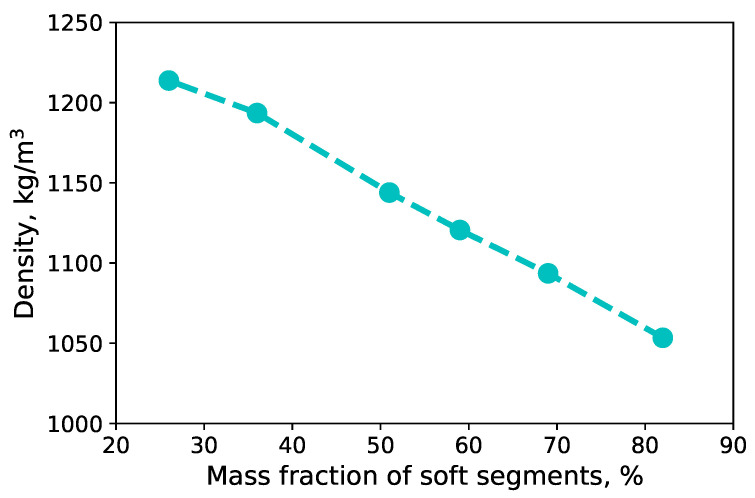
Density of TPU as function of mass fraction of soft segments at normal conditions.

For an investigation of the influence of the content of soft segments on the density of TPU, the density of each simulated system was calculated. It was observed that the density decreases linearly with an increase in the mass fraction of soft segments (see Figure 7). For comparison, the density of TPU depending on its chemical structure is in the range 1.1 g/${\mathrm{cm}}^{3}$ ... 1.21 g/${\mathrm{cm}}^{3}$[48] (1.2 g/${\mathrm{cm}}^{3}$[49], 1.11 g/${\mathrm{cm}}^{3}$[50], ≈1.2 g/${\mathrm{cm}}^{3}$ [51]).

**Figure 8:**
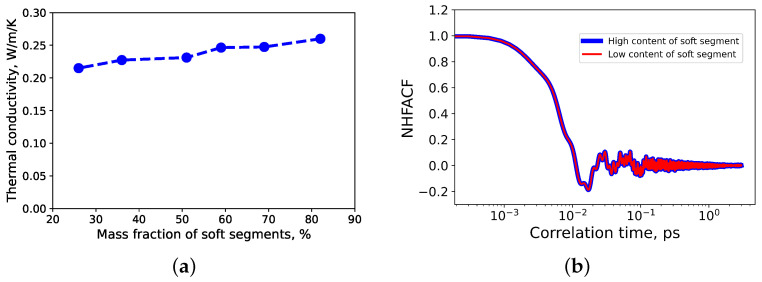
(**a**) The thermal conductivity of TPU as function of mass fraction of soft segments; (**b**) normalized heat flux autocorrelation functions (NHFACF) of high content (blue) and low content (red) of thermoplastic polyurethane.

A similar tendency was observed for the thermal conductivity of TPU: it increases linearly with an increase in the mass fraction of soft segments (see Figure 8a). The normalized heat flux autocorrelation functions of high-content and low-content thermoplastic polyurethane are shown in Figure 8b). The first minimum of the normalized heat flux autocorrelation function (see Figure 8b) is located roughly at 17 fs, which corresponds to a wave number $\bar{\lambda }$≈ 312 ${\mathrm{cm}}^{-1}$. It agrees well with other MD simulations of polymers, where it was revealed, that at normal conditions, the heat in polymers is mainly transferred by low frequency phonons [6,34,52]. They are caused by deformations of polymeric chains. For comparison, the thermal conductivity of TPU from experiments at normal conditions is found to be roughly 0.21 W/m/K [53].

## 4. Conclusions

Two main approaches (EMD and NEMD simulations) to predicting the thermal conductivity of natural rubber are presented. It was shown that both methods possess different advantages with respect to size effects of the systems and derived properties. The EMD method is less affected by size effects than the NEMD method. Moreover, thermal conductivities of silicone rubber were obtained as a function of the degree of crosslinking. The thermal conductivity increases with an increase in the degree of crosslinking. These results are in very good agreement with data in the literature. In addition, the thermal conductivity of TPU was derived as a function of the mass fraction of soft segments. The thermal conductivity of TPU almost linearly increases with an increase in the number of soft segments. From am analysis of normalized heat flux autocorrelation functions, it was revealed that heat in the polymers is mainly transferred by low-frequency phonons. Both methods (EMD and NEMD simulations) deliver an appropriate quantitative description of those complex polymeric structures.

## Figures and Tables

**Table 1:** Number of soft segments in one chain. Every chain consists of 40 segments.

Structure 1	Structure 2	Structure 3	Structure 4	Structure 5	Structure 6
30	33	36	37	38	39
